# Incidence and Predictors of Right Ventricular Reverse Remodeling in Patients with Transthyretin Amyloid Cardiomyopathy Treated with Tafamidis

**DOI:** 10.3390/biomedicines13051211

**Published:** 2025-05-16

**Authors:** Nicoleta Nita, Dominik Felbel, Michael Paukovitsch, Felix von Sanden, Elene Walter, Rima Melnic, Wolfgang Rottbauer, Dominik Buckert, Johannes Mörike

**Affiliations:** Department of Internal Medicine II, University Medical Center, 89081 Ulm, Germany; dominik.felbel@uniklinik-ulm.de (D.F.); michael.paukovitsch@uniklinik-ulm.de (M.P.); felix.vonsanden@uniklinik-ulm.de (F.v.S.); elene.walter@uniklinik-ulm.de (E.W.); rima.melnic@uniklinik-ulm.de (R.M.); wolfgang.rottbauer@uniklinik-ulm.de (W.R.); dominik.buckert@uniklinik-ulm.de (D.B.); johannes.moerike@uniklinik-ulm.de (J.M.)

**Keywords:** right ventricular reverse remodeling in ATTR-CM, right ventricular strain in ATTR-CM, RV-PA coupling in tafamidis-treated ATTR-CM, predictors of reverse remodeling in ATTR-CM

## Abstract

**Background/Objectives**: In patients with transthyretin amyloid cardiomyopathy (ATTR-CM), the effect of tafamidis on right ventricular (RV) dysfunction has been poorly investigated. The purpose of this study was to evaluate the effect of tafamidis on RV free wall global longitudinal strain (RV FW-GLS) and right ventricular and pulmonary artery (RV-PA) coupling over 12 months of treatment. **Methods**: Ninety-three patients with ATTR-CM treated with 61 mg of tafamidis daily who underwent multimodality imaging evaluation at baseline by cardiovascular magnetic resonance (CMR) and speckle-tracking echocardiography were retrospectively studied. The 12-month follow-up included an echocardiographic assessment of RV FW-GLS and RV-PA coupling. RV reverse remodeling was defined as a >10% improvement in RV FW-GLS and/or in RV-PA coupling from baseline. RV-PA coupling was assessed using the tricuspid annular plane systolic excursion/ pulmonary artery systolic pressure (TAPSE/PASP) ratio. **Results**: Over 12 months of tafamidis treatment, RV reverse remodeling was documented in 22.6% of patients. In these patients, RV FW-GLS improved significantly from 14.5 ± 2.1% to 17.3 ± 2%, *p* < 0.001, whereas the TAPSE/PASP ratio improved from 0.42 ± 0.05 mm/mmHg to 0.54 ± 0.07 mm/mmHg, *p* = 0.001. Patients who experienced RV reverse remodeling were at an earlier stage of disease prior to tafamidis treatment with less dilated RV and less severe RV-PA uncoupling (TAPSE/PASP ratio: 0.43 ± 0.06 mm/mmHg vs. 0.39 ± 0.06 mm/mmHg, *p* = 0.040). CMR-derived baseline RV end-systolic volume (HR 0.83, 95% CI 0.73–0.94, *p* = 0.005) and NT-proBNP (HR 0.989, 95% CI 0.988–0.999, *p* = 0.024) were the strongest independent predictors of RV reverse remodeling, followed by PASP (HR 0.82, 95% CI 0.69–0.98, *p* = 0.030). **Conclusions**: Patients with ATTR-CM treated with tafamidis at an earlier stage of the disease experienced RV reverse remodeling with significant improvement in RV FW-GLS and RV-PA coupling.

## 1. Introduction

Transthyretin amyloid cardiomyopathy (ATTR-CM) is a prevalent and emerging cause of heart failure that can eventually lead to cardiovascular death [1]. Advances in cardiac imaging have facilitated the diagnosis of ATTR-CM worldwide over the last decade, and recent studies from Germany report a significant and steep increase in both the prevalence (from 15.5 to 47.6 per 100,000 person-years) and incidence (from 4.8 to 11.6 per 100,000 person-years) of ATTR-CM [2]. Patients with ATTR-CM often develop right ventricular (RV) dysfunction even at early disease stages, either due to amyloid deposition in the RV or as a consequence of postcapillary pulmonary hypertension associated with chronically elevated left ventricular (LV) filling pressures [3]. Recent research using single-photon emission tomography imaging showed either a focal or diffuse RV uptake of bone tracers in 100% of patients with newly diagnosed ATTR-CM, and the extent of RV involvement was independently associated with increased mortality across all forms of ATTR-CM [4]. Other relevant mechanisms of RV dysfunction in ATTR-CM relate to RV ischemia, as recent positron emission tomography studies have shown significantly reduced RV myocardial blood flow associated with poor outcomes [5], but also with autonomic dysfunction, which is a common complication in ATTR-CM, mainly due to direct amyloid deposition in the autonomic nervous system [6]. RV free wall global longitudinal strain (RV FW-GLS) and right ventricle and pulmonary artery (RV-PA) coupling have emerged as robust tools to evaluate RV dysfunction and have been reported as important prognostic factors in patients with ATTR-CM [7,8]. Tafamidis is currently the only drug approved for both wild-type and hereditary ATTR-CM and has demonstrated a reduction in all-cause mortality and cardiovascular-related hospitalizations. Tafamidis selectively binds to transthyretin and stabilizes the tetramer, slowing monomer formation, misfolding, and amyloid deposition in the myocardial extracellular space [9]. However, the effect of tafamidis on RV dysfunction and its potential to reverse RV adverse remodeling has been poorly investigated. The purpose of this study was to assess the incidence and the predictors of RV reverse remodeling, which is defined as an improvement in RV FW-GLS or RV-PA coupling, in a contemporary cohort treated with tafamidis, using a comprehensive multimodality imaging evaluation with cardiovascular magnetic resonance (CMR) and speckle-tracking echocardiography.

## 2. Materials and Methods

This retrospective study included patients with ATTR-CM who were treated with tafamidis at the Amyloidosis Centre of the University Hospital of Ulm between April 2020 and January 2024 after approval in Germany. Only patients with a complete 1-year follow-up evaluation including echocardiography were included in this analysis. Demographic and clinical data, medical history, and follow-up data were retrospectively collected from the electronic medical records of the University Hospital of Ulm. This study was approved by the ethical board of the University of Ulm and complied with the Declaration of Helsinki. Informed consent was waived due to the retrospective design of the study. ATTR-CM was diagnosed according to current guidelines either by endomyocardial biopsy and the immunohistochemical classification of amyloid or by bone scintigraphy in the absence of monoclonal gammopathy in serum and urine immunofixation [10]. Except for patients without a complete echocardiographic follow-up, there were no exclusion criteria for patients in this study. Patients with severe tricuspid regurgitation were not excluded from this analysis. Before starting treatment with tafamidis, all patients included in this analysis underwent clinical and laboratory assessment, including New York Heart Association (NYHA) functional class, cardiac biomarkers (NT-proBNP and Troponin T), estimated glomerular filtration rate (eGFR), and multimodality imaging with transthoracic echocardiography and CMR. The disease stage at baseline was determined using the National Amyloidosis Centre (NAC) staging system.

### 2.1. Follow-Up and Definition of RV Reverse Remodeling

Follow-up included clinical, laboratory, and echocardiographic evaluation at 12 months after the initiation of tafamidis. RV reverse remodeling was defined as an improvement in RV FW-GLS or RV-PA coupling, or both, after 12 months of treatment with tafamidis. A relevant improvement in RV FW-GLS or RV-PA coupling was defined as a relative change of more than 10% from baseline, in accordance with previous research [11,12].

### 2.2. Echocardiographic Measurements

Echocardiographic measurements were performed by trained cardiac sonographers in agreement with the European Association of Cardiovascular Imaging recommendations [13]. Tricuspid annular plane systolic excursion (TAPSE) was tracked in the RV-focused apical four-chamber view using M-mode echocardiography. Pulmonary artery systolic pressure (PASP) was calculated as follows: 4 × [peak velocity of TR]2 + [estimated right atrial pressure]. Speckle-tracking RV FW-GLS was computed from the RV-focused apical views in accordance with the task force recommendations [14], excluding the interventricular septum, to detect the extension of the ATTR-CM into the RV. All measurements were performed using the Tom Tec Imaging System, TTA2.30.01 (Munich, Germany).

### 2.3. CMR Assessment

Patients underwent CMR examination using a protocol designed for cardiac amyloidosis on a clinical 1.5 Tesla scanner (Achieva, Philips Healthcare, Best, The Netherlands). Multi-slice b-SSFP Cartesian sequences and retrospective ECG gating were performed to cover the entire cardiac cycle with 32 phases and the following acquisition parameters: temporal resolution of 30 ms, repetition time of 2.42 ms, echo time of 1.2 ms, flip angle of 60°, field of view of 380 × 380 mm^2^, in plane resolution of 1.4 × 1.4 mm^2^, and slice thickness of 8 mm. Ventricular late gadolinium enhancement (LGE) acquisitions were performed in all subjects using an extracellular contrast agent (Dotarem^®^, Guerbet, Villepinte, France) and segmented inversion-recovery gradient sequences in the long and short axis according to the recommendations of the Society for Cardiac Magnetic Resonance [15]. The study protocol included a modified look-locker sequence in a 5(3)3 scheme for T1 mapping before and after the application of the contrast agent.

All CMR measurements, including volumetry, RV FW-GLS, LV GLS, parametric mapping, and LGE assessment, were performed using CVI42 software, v6.0 (Circle Cardiovascular Imaging Inc., Calgary, AB, Canada).

Inter-observer and intra-observer reproducibility analyses of RV strain measurements were performed in 25 randomly selected individuals and showed good results (Kappa coefficients of 0.76 and 0.74, respectively).

### 2.4. Statistics

Statistical analyses were performed with IBM SPSS Statistics, version 29.0 (IBM Corporation, Armonk, NY, USA). Continuous variables with normal distribution were expressed as mean ± standard deviation (SD) or median with interquartile range (IQR) for non-normal distribution. Categorical variables were expressed as absolute numbers and percentages. Comparisons between subgroups were performed using a t-test or the Mann–Whitney U test for continuous variables or the Chi-square test for categorical variables. The area under the curve (AUC) was determined by receiver operating characteristic (ROC) analysis to determine predictors of RV reverse remodeling. Optimal cut-off values for relevant parameters having an AUC above 0.7 were generated from the ROC analysis using the Youden threshold. Multivariable logistic regression analysis was performed to assess the influence of relevant parameters on RV reverse remodeling. The algorithm was applied to all potentially relevant variables, including parameters from univariate logistic regression analysis with *p* < 0.10. Collinearity between parameters was analyzed using variance inflation factors. A two-tailed *p* < 0.05 was considered statistically significant.

## 3. Results

### 3.1. Patient Characteristics

**Figure 1:**
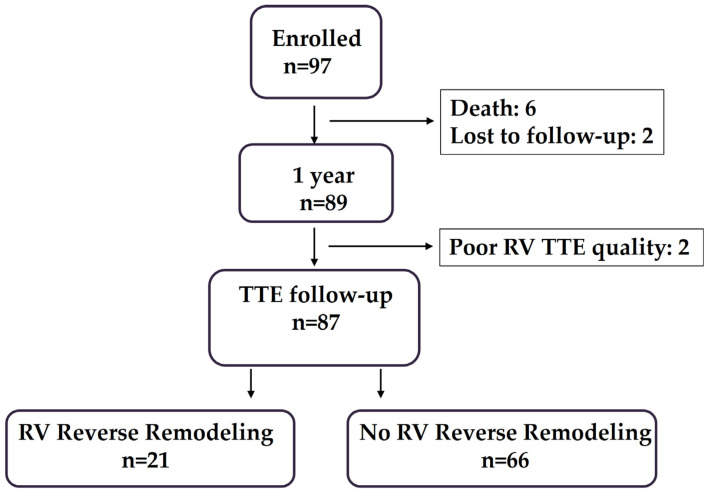
Illustration of enrollment of patients with ATTR-CM treated with tafamidis and followed up at 1 year. TTE, transthoracic echocardiography; RV, right ventricle.

Of a total of 97 patients with ATTR-CM treated with tafamidis, 4 patients were excluded from the analysis: 2 patients were lost to follow-up, and 2 patients had poor echocardiographic quality of RV strain measurements at follow-up, as shown in Figure 1.

The mean age of the remaining study patients was 80 ± 6.5 years, 80.6% were male, and 75 patients had wild-type ATTR-CM. Most patients were in NAC stage 1 (41.9%) and NAC stage 2 (35.5%) and presented with a high prevalence of comorbidities, as shown in Table 1. The majority of patients were receiving optimal medical therapy for heart failure, including angiotensin receptors–neprilysin inhibitors/angiotensin-converting enzyme inhibitors/angiotensin receptor blockers (57%) and sodium–glucose cotransporter 2 (SGLT2) inhibitors (59.1%), with no significant differences between patients who developed RV reverse remodeling and those who did not. Patients with RV reverse remodeling had significantly lower NT-proBNP levels at baseline compared to patients with no reverse remodeling (2053 ± 784 pg/mL vs. 3097 ± 1066 pg/mL, *p* < 0.001), whereas eGFR was not significantly different between patients with and without RV reverse remodeling.

### 3.2. Results of Multimodality Imaging

The baseline results of CMR and echocardiography are depicted in Table 2. CMR assessment showed a mean LVEF of 43 ± 4%, a mean RVEF of 49 ± 5%, and a mean extracellular volume (ECV) of 51 ± 6.4%, with no significant differences between patients who developed RV reverse remodeling and those who did not. Patients without RV reverse remodeling had significantly higher RV end-systolic volumes (RV-ESVs) (42 ± 5.6 mL/m^2^ vs. 35 ± 5.1 mL/m^2^, *p* < 0.001) and a higher prevalence of severe tricuspid regurgitation (22.7% vs. 4.8%, *p* = 0.064) at baseline compared to patients with RV reverse remodeling. Mean RV FW-GLS at baseline was 14.9 ± 2.7%, without significant differences between patients with RV reverse remodeling and those without. Patients without RV reverse remodeling had a significantly higher PASP (43 ± 4.2 mmHg vs. 40 ± 3.6 mmHg, *p* = 0.004) and a lower TAPSE/PASP ratio (0.39 ± 0.06 mm/mmHg vs. 0.43 ± 0.05 mm/mmHg, *p* = 0.040) compared to patients with RV reverse remodeling.

### 3.3. RV Reverse Remodeling at Follow-Up

At the 1-year follow-up, RV reverse remodeling was documented in 21 patients (22.6%), all of whom had a significant improvement in mean RV FW-GLS from 14.5 ± 2.1% to 17.3 ± 2%, *p* < 0.001. In the 66 patients without RV reverse remodeling, RV FW-GLS did not change at the 12-month follow-up (15.2 ± 2.8% vs. 15.1 ± 2.6%). Seventeen patients (18.3%) experienced significant improvement in the TAPSE/PASP ratio from 0.42 ± 0.05 mm/mmHg to 0.54 ± 0.07 mm/mmHg, *p* = 0.001. None of the patients experienced isolated improvement in the TAPSE/PASP ratio, as patients with an improvement in the TAPSE/PASP ratio also showed improvement in RV FW-GLS. During the follow-up period of 12 months, 6 patients (6.5%) died from cardiac causes, hospitalization due to decompensated heart failure was documented in 18 patients (19.4%), and 2 patients experienced sustained ventricular tachycardia. None of the patients underwent cardiac surgery during the follow-up period.

### 3.4. Predictors of RV Reverse Remodeling

**Figure 2:**
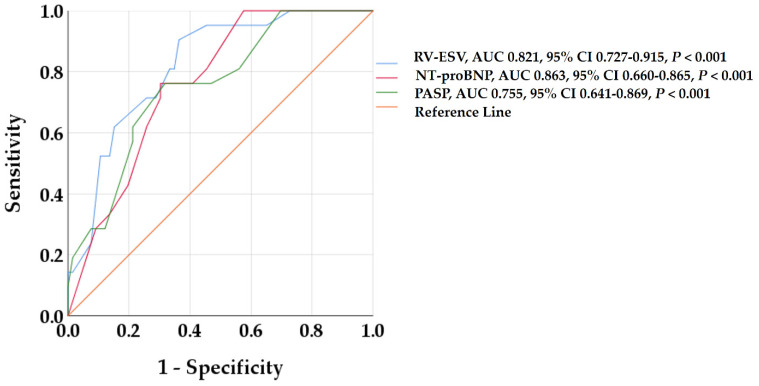
Receiver operating characteristic curves to detect predictors of RV reverse remodeling. RV-ESV, NT-proBNP, and PASP showed the highest AUC among all baseline variables. AUC, area under the curve; CI, confidence interval; NT-proBNP, B-type natriuretic peptide; PAPS, pulmonary artery systolic pressure; RV-ESV, right ventricular end-systolic volume.

In the ROC analysis, RV-ESV, NT-proBNP, and PASP showed the best predictive value for RV reverse remodeling (Figure 2). Optimized cut-off values of 41.5 mL/m^2^ for RV-ESV, 2490 pg/mL for NT-proBNP, and 41 mmHg for PASP were identified as predictive of RV reverse remodeling.

Table 3 and Table 4 summarize the results of univariate and multivariate logistic regression analysis, including cardiac imaging and clinical variables.

In multivariable logistic regression analysis, baseline RV-ESV and NT-proBNP were the strongest predictors of RV reverse remodeling, followed by the presence of pulmonary hypertension, as shown in Table 4.

## 4. Discussion

In this study, we comprehensively evaluated the morphologic and functional patterns of RV dysfunction using a multimodality imaging approach and assessed the incidence and predictors of RV reverse remodeling in a contemporary cohort with ATTR-CM treated with tafamidis. Our study shows the following findings: (1) RV reverse remodeling with an improvement in RV FW-GLS and RV-PA coupling was documented in 22.6% of patients during 1 year of treatment with tafamidis; (2) patients treated with tafamidis at an earlier stage of disease are more likely to experience RV reverse remodeling, as RV-ESV, NT-proBNP, and the degree of pulmonary hypertension were the main independent predictors of RV reverse remodeling.

Currently, the concept of RV reverse remodeling is not standardized. Several echocardiographic and CMR parameters have been previously investigated to assess RV reverse remodeling, including RV end-diastolic area, LV eccentricity index in patients with pulmonary hypertension treated with specific vasodilator agents [16], or RV-ESV in patients undergoing transcatheter valve replacement for tricuspid regurgitation [17]. However, none of these parameters have been validated in larger trials, and RV reverse remodeling in patients with ATTR-CM treated with tafamidis has been barely investigated, making the interpretation of our results difficult.

RV dysfunction in ATTR-CM is complex and is caused not only by amyloid accumulation extending into the RV and pulmonary vascular infiltration–related precapillary pulmonary, but also as a consequence of increased preload associated with tricuspid regurgitation [18]. Moreover, increased RV afterload due to elevated LV filling pressures, left atrial cardiomyopathy, mitral regurgitation, and postcapillary pulmonary hypertension plays a significant role in RV dysfunction. Furthermore, positron emission tomography perfusion imaging has shown that RV perfusion is reduced in proportion to RV dysfunction and pulmonary artery pressure in patients with ATTR-CM [5]. RV FW-GLS reflects, in a sensitive manner, the RV dysfunction independently of and earlier than RV hypertrophy and dilatation. Patients in this real-world cohort showed mildly reduced CMR-derived RVEF but significantly reduced RV FW-GLS prior to tafamidis initiation. We report a significant improvement in RV FW-GLS in 22.6% of patients at the 1-year follow-up. Recently, Nagai et al. reported an improvement in RV FW-GLS from −17.8 ± 7.9% at baseline to −24.5 ± 9.1% at 12 months in a small cohort of 33 patients with ATTR-CM treated with 80 mg of tafamidis daily. Our results show a less pronounced improvement in RV FW-GLS; however, patients in our study started tafamidis at a more advanced stage of disease, highlighting the importance of timely treatment.

The TAPSE/PASP ratio is a non-invasively measured index of RV–pulmonary circulation coupling that correlates with invasively assessed RV systolic elastance and pulmonary arterial elastance [19,20]. The coupling between the RV and its afterload provides a more comprehensive evaluation of RV performance than TAPSE or PASP alone [21].

To date, only Tomasoni et al. have demonstrated the prognostic value of the TAPSE/PASP ratio in a large cohort of patients with ATTR-CM. A TAPSE/PASP ratio <0.45 mm/mmHg was independently associated with all-cause death and hospitalization for heart failure [7]. However, the improvement in the TAPSE/PASP ratio in patients with ATTR-CM treated with tafamidis has not been previously investigated. We report a significant improvement in the TAPSE/PASP ratio in 18.3% of patients after 12 months of tafamidis treatment. Notably, none of the patients experienced isolated improvement in the TAPSE/PASP ratio without improvement in RV FW-GLS, suggesting that the improvement in RV strain precedes the optimized RV-PA coupling in tafamidis-treated patients with ATTR-CM.

Multivariable regression analysis identified the presence of extensive RV dilation, elevated NT-proBNP levels, and the degree of pulmonary hypertension as the strongest independent predictors of RV reverse remodeling, underlining that the timely initiation of tafamidis is key to disease progression and treatment response.

### Strengths and Limitations

This study has several limitations. First, it is a retrospective, observational study with a relatively small population size and a limited follow-up duration of 12 months. However, our study is the largest to analyze RV reverse remodeling and the first to report improvement in RV-PA coupling in tafamidis-treated patients with ATTR-CM. Therefore, our results should be interpreted as a pilot investigation that warrants external validation in larger dedicated studies. Importantly, the prognostic implications of RV reverse remodeling remain to be elucidated in long-term follow-ups. Finally, it is difficult to distinguish the effect of tafamidis from the effect of optimal medical therapy, as the widespread use of guideline-directed medical therapy may have contributed significantly to RV remodeling.

## 5. Conclusions

Patients with ATTR-CM treated with tafamidis experience RV reverse remodeling with significant improvement in RV FW-GLS and RV-PA coupling. The presence of extensive RV dilation, elevated NT-proBNP levels, and the degree of pulmonary hypertension predicts RV reverse remodeling and highlights the importance of the timely initiation of tafamidis before myocardial and pulmonary adverse remodeling becomes irreversible. Our results emphasize that a comprehensive assessment of RV function should be routinely performed in patients with ATTR-CM prior to the initiation of tafamidis to allow for appropriate risk stratification and predictions of therapeutic response.

## Figures and Tables

**Table 1:** Baseline characteristics.

Variable	No RV Reverse Remodeling (N = 66)	RV Reverse Remodeling (N = 21)	*p* Value
Age	80 ± 7	78 ± 6	0.166
Male gender (%)	56(84.8)	15(71.4)	0.167
NYHA functional class (%)			
I	0	0	
II	45(68.2)	18(85.7)	0.117
III	21(31.8)	3(14.3)	0.117
IV	0	0	
NAC stage			
I	24(36.4)	10(47.6)	0.357
II	25(37.9)	9(42.9)	0.986
III	17(25.8)	3(14.3)	0.276
Prior HF hospitalization (%)	30(45.5)	8(38.1)	0.554
Coronary artery disease (%)	36(54.5)	8(38.1)	0.189
Atrial fibrillation (%)	46(69.7)	13(61.9)	0.506
NT-proBNP (pg/mL)	3097 ± 1066	2053 ± 784	<0.001
Troponin T (ng/L)	30 ± 14	27 ± 13	0.497
eGFR (ml/min/1.73 cm^2^)	39 ± 7	40 ± 6	0.597
ACEi/ARB	32(48.5)	9(42.9)	0.653
ARNI	21(31.8)	9(42.9)	0.354
*β*-blocker	45(68.2)	15(71.4)	0.779
MRA	40(60.6)	15(71.4)	0.370
SGLT2 inhibitor	39(59.1)	11(52.4)	0.588
Loop diuretics	47(71.2)	11(52.4)	0.111

Values are presented as n(%) or mean ± SD. ACEi, angiotensin-converting enzyme inhibitor; ARB, angiotensin receptor blocker; ARNI, angiotensin receptor–neprilysin inhibitor; NT-proBNP, B-type natriuretic peptide; eGFR, estimated glomerular filtration rate; HF, heart failure; MRA, mineralocorticoid receptor antagonist; N, number of patients; NAC, National Amyloidosis Centre; NYHA, New York Heart Association; SGLT2, sodium–glucose cotransporter 2.

**Table 2:** Results of multimodality imaging at baseline.

Variable	No RV Reverse Remodeling (N = 66)	RV Reverse Remodeling (N = 21)	*p* Value
CMR			
ECV (%)	52 ± 6.1	49 ± 7	0.124
LVEF (%)	43 ± 4	43 ± 5	0.553
LV GLS (%)	11 ± 2	10 ± 1.9	0.659
IVSd (mm)	18 ± 1.5	18 ± 1.8	0.837
RVEF (%)	50 ± 5	49 ± 6	0.394
RV-ESV (ml/m^2^)	42 ± 5.6	35 ± 5.1	<0.001
RV FW-GLS (%)	15.4 ± 2.9	14.4 ± 2.1	0.178
Echocardiography			
RV FW-GLS (%)	15.2 ± 2.8	14.5 ± 2.1	0.302
TAPSE (cm)	1.51 ± 0.11	1.48 ± 0.15	0.269
PASP (mmHg)	43 ± 4.2	40 ± 3.6	0.004
TAPSE/PASP (mm/mmHg)	0.39 ± 0.06	0.43 ± 0.06	0.040
Severe Tricuspid regurgitation	15(22.7)	1(4.8)	0.064

Values are presented as n(%) or mean ± SD. CMR, cardiovascular magnetic resonance; ECV, extracellular volume; IVSd, interventricular septal thickness in diastole; LVEF, left ventricular ejection fraction; LV GLS, left ventricular global longitudinal strain; N, number of patients; RV-ESV, right ventricular end-systolic volume; PASP, pulmonary artery systolic pressure; RVEF, right ventricular ejection fraction; RV FW-GLS, right ventricular free wall global longitudinal strain; TAPSE, tricuspid annular plane systolic excursion.

**Table 3:** Results of univariate logistic regression analysis for the prediction of RV reverse remodeling in patients with ATTR-CM.

Variables	HR (95% CI)	*p* Value
Age	0.95 (0.89–1.02)	0.172
Gender	0.45 (0.14–1.42)	0.174
NYHA class III	2.80 (0.74–10.56)	0.128
NAC stage I	1.59 (0.59–4.29)	0.159
NAC stage III	0.48 (0.13–1.83)	0.284
NT-proBNP (pg/mL)	0.989 (0.988–0.999)	<0.001
Loop diuretics	0.44 (0.24–1.85)	0.136
SGLT2 inhibitor	0.76 (0.28–2.04)	0.589
PASP, mmHg	0.82 (0.72–0.95)	0.006
RV-ESV, ml/m^2^	0.80 (0.72–0.89)	<0.001
RVFW-GLS (%)	0.88 (0.74–1.06)	0.178
TAPSE, cm	1.07 (0.70–16.39)	0.379
Global ECV (%)	0.94 (0.87–1.02)	0.125
Severe tricuspid regurgitation	1.76 (0.21–1.37)	0.096
RVEF (%)	1.04 (0.95–1.15)	0.391

ECV, extracellular volume; NAC, National Amyloidosis Centre; NT-proBNP, N-terminal pro B-type natriuretic peptide; NYHA, New York Heart Association; PASP, pulmonary artery systolic pressure; RVEF, right ventricular ejection fraction; RV-ESV, right ventricular end-systolic volume; RVFW-GLS, right ventricular free wall global longitudinal strain; SGLT2, sodium–glucose cotransporter 2; TAPSE, tricuspid annular plane systolic excursion.

**Table 4:** Multivariable predictors of RV reverse remodeling in ATTR-CM.

Variable	HR (95% CI)	*p* Value
Severe tricuspid regurgitation	3.78 (0.39–37)	0.251
RV-ESV (ml/m^2^)	0.83 (0.73–0.94)	0.005
NT-proBNP (pg/mL)	0.989 (0.988–0.999)	0.024
PASP (mmHg)	0.82 (0.69–0.98)	0.030

Multivariable models based on cause-specific logistic regression analyses. NT-proBNP, B-type natriuretic peptide; PASP, pulmonary artery systolic pressure; RV-ESV, right ventricular end-systolic volume.

## Data Availability

The data underlying this article will be shared upon reasonable request to the corresponding author. The data are not publicly available due to data privacy laws.

## Abbreviations

The following abbreviations are used in this manuscript:

ATTR-CM	Transthyretin amyloid cardiomyopathy
CMR	Cardiovascular magnetic resonance
LGE	Late gadolinium enhancement
LVEF	Left ventricular ejection fraction
NAC	National Amyloidosis Centre
NYHA	New York Heart Association
PASP	Pulmonary artery systolic pressure
RV	Right ventricle
RVEF	Right ventricular ejection fraction
RV-ESV	Right ventricle end-systolic volume
RV FW-GLS	RV free wall global longitudinal strain
RV-PA	Right ventricular to pulmonary artery
TAPSE	Tricuspid annular plane systolic excursion
